# Supplementary material for: What makes a pair bond in a Neotropical primate: female and male contributions
Source: R Soc Open Sci. 2020 Jan 15;7(1):191489. doi: 10.1098/rsos.191489 (PMC7029894; doi:10.1098/rsos.191489)
Supplement: Group compositions and birthdates [file rsos191489supp2.docx]

Table S1. Group compositions indicated for the respective study periods, total and focal observation hours collected, and dates of birth of infants for 7 studied groups. AM – adult male, AF – adult female, Sub – subadult (M: male; F: female), Juv – juvenile, Inf – infant. Sex could not be determined for juveniles and infants due to small genital size.

| Group | Study period | Total sampling time, h | Focal sampling time, h | AM | AF | Sub | Juv | Inf | Infant date of birth^*^ |
| --- | --- | --- | --- | --- | --- | --- | --- | --- | --- |
| 1 | Jun – Dec 2017 | 482.4 | 139.4 | 1 | 1 | 1 (M) | 1 | 0 | - |
|  | Sep – Dec 2018 |  |  | 1 | 1 | 1 (M) | 1 | 1 | 05.06.2018 |
| 2 | Sep – Oct 2017 | 479.9 | 55.3 | 1 | 1 | 0 | 0 | 0 | - |
|  | Jul 2018, Oct – Dec 2018 |  |  | 1 | 1 | 0 | 0 | 1 | 26.10.2018 |
| 3 | Oct – Dec 2017 | 393.6 | 20.8 | 1 | 1 | 0 | 1 | 1 | 09.2018 |
|  | Jun 2018 |  |  | 1 | 1 | 1 (M) | 1 | 0 | - |
| 4 | Jun – Jul 2018, Sep – Oct 2018 | 387.2 | 86.0 | 1 | 1 | 0 | 1 | 0 | - |
| 5 | Aug – Oct 2018 | 294.8 | 38.3 | 1 | 1 | 0 | 1 | 0 | - |
| 6 | Aug – Oct 2017 | 520.3 | 38.4 | 1 | 1 | 1 (M) – 2 (M ,F) | 1 | 1 | 05.10.2017 |
|  | Jul 2018 |  |  | 1 | 1 | 2 (M, F) | 1 | 0 | - |
| 7 | Aug 2018, Nov 2018 | 192.6 | 12.7 | 1 | 1 | 0 | 0 | 1 | 01.11.2018 |

* We estimated an infant’s date of birth as the midpoint between the dates when a group was last seen without and first seen with an infant. The difference between these dates varied between 0 and 26 days. For Group 3, the date of birth could only be estimated within a month based on body size as the group already had an infant when we started to follow it. However, at the end of the study period the infant still did not begin to travel independently, indicating that it was younger than 4 months (1,2).

1. Fragaszy DM, Schwarz S, Shimosaka D. Longitudinal observations of care and development of infant titi monkeys (*Callicebus moloch*). Am J Primatol. 1982;doi:2:191–200. 10.1002/ajp.1350020207

2. Wright PC. Biparental care in *Aotus trivirgatus* and *Callicebus moloch*. In: Small MF, editor. Female primates: studies by women primatologists. New York, NY: Alan R. Liss; 1984. p. 59–75.
