# Supplementary material for: What makes a pair bond in a Neotropical primate: female and male contributions
Source: R Soc Open Sci. 2020 Jan 15;7(1):191489. doi: 10.1098/rsos.191489 (PMC7029894; doi:10.1098/rsos.191489)
Supplement: Model results [file rsos191489supp3.docx]

Table S2. Results of the models on (1) rates of proximity and affiliation; (2) grooming reciprocity between pair mates. Indicated are estimates, standard errors, confidence intervals, results of likelihood ratio tests, and the range of estimates obtained when dropping levels of random effects one at a time.

| **Term** | **Estimate** | **SE** | **Lower CI** | **Upper CI** | **χ2** | **df** | **P-value** | **min** | **max** |
| --- | --- | --- | --- | --- | --- | --- | --- | --- | --- |
| **Model 1: rates of proximity and affiliation**  The model was fairly stable; maximum Variance Inflation Factor: 1.517; dispersion parameter = 0.996 | | | | | | | | | |
| Intercept | -0.378 | 0.123 | -0.618 | -0.138 | - | - | - | -0.484 | -0.230 |
| Infant presence ^(1)^ | -1.019 | 0.234 | -1.495 | -0.543 | 16.524 | 1 | 0.000 | -1.102 | -0.801 |
| Group size ^(2)^ | -0.325 | 0.201 | -0.720 | 0.069 | 3.759 | 1 | 0.053 | -0.410 | -0.142 |
| Rainfall ^(3)^ | 0.074 | 0.138 | -0.197 | 0.345 | 0.266 | 1 | 0.610 | 0.075 | 0.252 |
| **Model 2: grooming reciprocity**  The model was stable; maximum Variance Inflation Factor: 1.783; dispersion parameter = 0.789 | | | | | | | | | |
| Intercept | 0.128 | 0.132 | -0.130 | 0.387 | - | - | - | 0.054 | 0.193 |
| Infant presence ^(1)^ | 1.348 | 0.352 | 0.657 | 2.039 | 15.403 | 1 | 0.000 | 1.309 | 1.457 |
| Group size ^(2)^ | -0.038 | 0.123 | -0.278 | 0.203 | 0.094 | 1 | 0.759 | -0.212 | 0.037 |
| Rainfall ^(3)^ | -0.329 | 0.152 | -0.627 | -0.032 | 3.919 | 1 | 0.048 | -0.406 | -0.201 |

1. Dummy coded with absence of infant being the reference category
2. Z-transformed; mean ± sd of the original value: 3.216 ± 0.877
3. Z-transformed; mean ± sd of the original value: 91.754 ± 79.121
