# Supplementary material for: What makes a pair bond in a Neotropical primate: female and male contributions
Source: R Soc Open Sci. 2020 Jan 15;7(1):191489. doi: 10.1098/rsos.191489 (PMC7029894; doi:10.1098/rsos.191489)
Supplement: Intergroup encounters [file rsos191489supp4.docx]

Table S3. Time and participation in the observed intergroup encounters. Individual participation could only be determined for the groups followed by the observers. AM – adult male, AF – adult female, SM – subadult male, SF – subadult female, Juv – juvenile. Rows in bold indicate the encounters initiated by males.

1. – indicates an encounter that did not have any clear initiator
2. On 11 September 2018, the subadult male dispersed from Group 1 and by 18 September established a new territory with an unknown (to the observers) female next to the home range of his natal group. Since then and until 7 December 2018, when we stopped following Group 1, we observed 6 intergroup encounters between Group 1 and the newly established pair (indicated here as Group 11)
3. We never observed Groups 4 and 5 engaging in intergroup encounters. Most likely, it is due to the absence of territory overlap with neighbouring groups; this notion is indirectly supported by the fact that we never observed any intergroup encounters in Group 1 until September 2018 (see (2))
4. Chasing data could not be collected because of the poor observation conditions

| Date | Time | Group followed/encounter with group ^(3)^ | Participants (from the group followed) | Initiator | AM calling | AM chasing | AF calling | AF chasing | Notes |
| --- | --- | --- | --- | --- | --- | --- | --- | --- | --- |
| **09.09.17** | **8:26-9:00** | **6/2** | **AM + SM** | **AM** | **yes** | **yes** | **no** | **no** | **AF and Juv did not participate** |
| **13.09.17** | **06:02-6:17** | **2/2** | **AM + AF** | **AM** | **yes** | **yes** | **yes** | **no** | **AM was the first to start and the last to stop calling** |
| 13.09.17 | 10:22-10:36 | 2/6 | AM + AF | – ^(1)^ | yes | yes | yes | no |  |
| 16.09.17 | 11:10-11:22 | 2/6 | AM + AF | – | yes | yes | yes | no |  |
| **21.06.18** | **08:46-8:50** | **6/3** | **AM + AF + SM + SF** | **AM** | **yes** | **yes** | **yes** | **no** | **AF and SF joined for calling only in the end of the encounter but did not participate in chasing** |
| **17.07.18** | **10:22-10:46** | **6/2** | **AM + AF + SM + SF + Juv** | **AM** | **yes** | **yes** | **yes** | **yes** | **AM was the first to start and the last to stop calling** |
| 19.07.18 | 8:50-8:53 | 2/3 | AM + AF | – | yes | yes | yes | no |  |
| 25.07.18 | 7:48-7:50 | 3/2 | AM + AF + SM | – | yes | yes | yes | no |  |
| 16.08.18 | 6:05-6:11 | 7/unhabituated group | AM + AF | – | yes | N/A ^(4)^ | yes | N/A |  |
| 17.08.18 | 7:00-7:02 | 7/unhabituated group | AM + AF | – | yes | N/A | yes | N/A |  |
| 18.09.18 | 5:38-5:55 | 1/11 | AM + AF + Juv | – | yes | N/A | yes | N/A |  |
| 18.09.18 | 6:31-6:46 | 1/11 | AM + AF | – | yes | N/A | yes | N/A |  |
| 18.09.18 | 7:31-7:58 | 1/11 | AM + AF + Juv | – | yes | N/A | yes | N/A | Juv called only for the last 10 min of the encounter |
| **19.09.18** | **6:33-7:15** | **1/11** | **AM + AF** | **AM** | **yes** | **yes** | **yes** | **no** |  |
| **12.10.18** | **10:31-11:15** | **1/11** | **AM + AF** | **AM** | **yes** | **yes** | **yes** | **no** | **AM called and chased SM from Group 11; AF from Group 1 and AF from Group 11 only called but did not chased** |
| 13.10.18 | 8:52-9:10 | 1/11 | AM + AF | – | yes | yes | yes | yes |  |
| 15.10.18 | 6:43-7:41 | 1/11 | AM + AF + Juv | – | yes | yes | yes | no |  |
| 15.10.18 | 8:59-9:07 | 1/11 | AM + AF | – | yes | yes | yes | no |  |
| **30.11.18** | **6:35-6:38** | **1/11** | **AM** | **AM** | **yes** | **yes** | **no** | **no** | **AM called and chased SM from Group 11; neither AF from Group 1 nor AF from Group 11 called or chased** |
| **07.12.18** | **9:56-10:16** | **2/3** | **AM + AF** | **AM** | **yes** | **yes** | **yes** | **no** | **AM was the first to start and the last to stop calling; AF only called and did not chase** |
| **11.12.18** | **6:17-6:22** | **2/3** | **AM + AF** | **AM** | **yes** | **yes** | **yes** | **no** | **AM was the first to start and the last to stop calling** |
